# Threat Anticipation in Pulvinar and in Superficial Layers of Primary Visual Cortex (V1). Evidence from Layer-Specific Ultra-High Field 7T fMRI

**DOI:** 10.1523/ENEURO.0429-19.2019

**Published:** 2019-12-09

**Authors:** Ai Koizumi, Minye Zhan, Hiroshi Ban, Ikuhiro Kida, Federico De Martino, Maarten J. Vaessen, Beatrice de Gelder, Kaoru Amano

**Affiliations:** 1 Center for Information and Neural Networks (CiNet), National Institute of Information and Communications Technology (NICT) and Osaka University, Osaka 565-0871, Japan; 2 Sony Computer Science Laboratories, Inc, Tokyo, 141-0022, Japan; 3 Graduate School of Media and Governance, Keio University, Kanagawa 252-0882, Japan; 4 Department of Cognitive Neuroscience, Maastricht University, 6229 EV Maastricht, The Netherlands; 5 Department of Computer Science, University College London, London WC1E 6EA, England

**Keywords:** 7 tesla fMRI, fearful face, pulvinar, threat perception, V1 cortical layer

## Abstract

The perceptual system gives priority to threat-relevant signals with survival value. In addition to the processing initiated by sensory inputs of threat signals, prioritization of threat signals may also include processes related to threat anticipation. These neural mechanisms remain largely unknown. Using ultra-high-field 7 tesla (7T) fMRI, we show that anticipatory processing takes place in the early stages of visual processing, specifically in the pulvinar and V1. When anticipation of a threat-relevant fearful face target triggered false perception of not-presented target, there was enhanced activity in the pulvinar as well as in the V1 superficial-cortical-depth (layers 1–3). The anticipatory activity was absent in the LGN or higher visual cortical areas (V2–V4). The effect in V1 was specific to the perception of fearful face targets and did not generalize to happy face targets. A preliminary analysis showed that the connectivity between the pulvinar and V1 superficial-cortical-depth was enhanced during false perception of threat, indicating that the pulvinar and V1 may interact in preparation of anticipated threat. The anticipatory processing supported by the pulvinar and V1 may play an important role in non-sensory-input-driven anxiety states.

## Abstract (precis)

The perceptual system gives priority to threat-relevant signals with survival value. In addition to the processing initiated by sensory inputs of threat signals, prioritization of threat signals may also include processes related to threat anticipation. These neural mechanisms remain largely unknown. Using ultra-high-field 7 tesla (7T) fMRI, we show that anticipatory processing takes place in the early stages of visual processing, specifically in the pulvinar and V1.

## Significance Statement

States of anxiety may typically be triggered by mere anticipation of threatening sensory inputs even without actual presentation. However, the mechanisms in which mere anticipation of threat modulates the processing of incoming sensory inputs remain poorly understood, partly because the neural mechanisms underlying anxiety are often examined by measuring the effects initiated by visual presentation of actual threat stimuli. This study addresses how anticipation of threat modulates our visual system in its earliest stages. Specifically, this study shows that activity in the pulvinar and V1 is modulated based on anticipation of threat signals (fearful faces), leading to false perception of anticipated-yet-not presented threat signals.

## Introduction

Fear and anxiety are core states of the organism and understanding these is a central issue for neuroscience. The importance of these psychological states is highlighted by findings that the visual system prioritizes inputs with high behavioral relevance (Bishop, 2007) such as threat signals from facial or bodily expression (Lanzetta and Orr, 1986; de Gelder, 2006; Koizumi et al., 2016). Fear is triggered by actual threat signals, but threat stimuli can also be anticipated and induce fear as is typically the case in anxiety (Grillon et al., 2017; Torrisi et al., 2018). The neural mechanisms underlying such anticipatory processing of threat, however, remain largely unknown.

We here hypothesize that when threat is anticipated, activity changes are seen in the early stage of visual processing in the pulvinar and V1. The literature postulates the pulvinar as a central relay, forwarding threat-relevant sensory inputs to other cortical and subcortical areas for quick evaluation and response (LeDoux, 1996; Pessoa and Adolphs, 2010; Tamietto and de Gelder, 2010; McFadyen et al., 2017). In addition to input-driven processing, the pulvinar is also known to receive inputs from higher cortex including prefrontal areas (Grieve et al., 2000; Bridge et al., 2016) presumably contributing to higher-level perceptual processing (Saalmann and Kastner, 2011; Kanai et al., 2015; Roth et al., 2016). In addition, we hypothesize that anticipatory processing of threat may involve the earliest stage of visual cortical hierarchy, V1. Studies using simple visual stimuli such as gratings have shown that V1 activity reflects not only the physically presented stimuli but also the subjective perception as in the case of false alarm (FA; Ress and Heeger, 2003; Pajani et al., 2015). Relatedly, a recent study has demonstrated that visual images that are not physically present, but are well expected from the surrounding scenes, can induce the expected-image-like activity in V1 (Muckli et al., 2015). This effect was observed specifically in its superficial layers, known to be modulated by the pulvinar in non-human primates (Shipp, 2003, 2007). These results suggest an active role of V1, especially its superficial cortical depth, in shaping visual perception in a top-down manner, at least when guided by some sensory inputs (Muckli et al., 2015).

**Figure 1.**
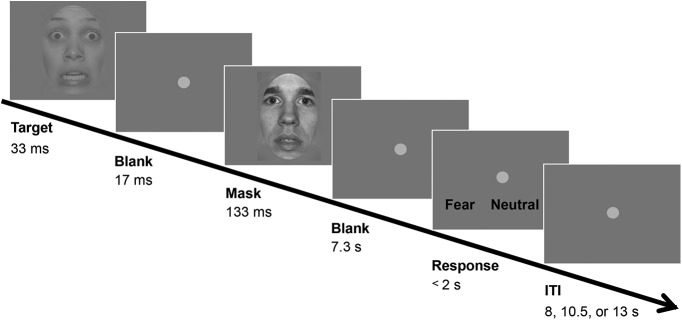
Design of the fearful face detection task. In each trial, either a fearful face target or a neutral face was presented briefly, followed by a mask consisting of a neutral face with a different identity than the target face. Participants responded whether they perceived a fearful target or neutral face by pressing the response key, which was randomly assigned trial-wise. The control task used happy face targets and neutral faces (images not shown), and otherwise identical procedures. ITI: intertrial interval. See also Extended Data Figure 1-1.

To examine the role of the pulvinar and V1 in threat anticipation, we designed an experiment where participants performed a simple task to detect a fearful face target (Fig. 1), which served as a social threat signal (Lanzetta and Orr, 1986; de Gelder, 2006). In a separate control session, a happy face detection task allowed us to examine whether the pulvinar and/or V1 contribute specifically to anticipation of threat or generalize to any salient target. Importantly, participants were informed which target would be presented before each session to build their anticipation of a given target (Pajani et al., 2015). Prior knowledge combined with weakened sensory input due to brief presentation, induced participants’ anticipation leading to more false percepts (Friston, 2005; Pajani et al., 2015). In each session, target faces (fearful or happy) and neutral faces were presented in each half of the trials in a randomized order. Participants falsely perceived either a fearful or happy face target when a neutral face was actually presented in ∼25% of the cases. We hypothesize that, unlike the trials where participants correctly perceive the presented fearful faces (i.e., HIT trials), the percept of a fearful or happy face in trials where a neutral face is presented (i.e., FA trials) cannot be explained by sensory input of the presented face but is instead likely caused by anticipatory processing. Although it is expected that participants’ anticipation for an upcoming threat-relevant fearful face target is noisy and fluctuates similarly across all trials, the proportion of trials where a higher level of anticipation contributes to the percept of a fearful face is likely to be larger among the FA than the HIT trials (Extended Data Fig. 1-1).

10.1523/ENEURO.0429-19.2019.f1-1 Extended Data Figure 1-1 Illustration of the differential contribution of anticipation for the HIT and FA trials. Given that there was no trial-wise cue to forecast the upcoming face stimulus (i.e., fearful or neutral), it is expected that anticipation for the fearful face target was similarly fluctuating across all the trials regardless of the actual type of face stimulus. That is, regardless of the presented face stimulus (fearful or neutral), there are likely to be similar proportions of trials with relatively higher level of anticipation (see inner red circle in the center). When comparing the HIT and FA trials, however, heightened anticipation is likely to contribute to a larger proportion of the FA trials relative to the HIT trials where sensory inputs of presented fearful face also contributed to the percept. This can be inferred when simply considering the proportion of HIT trials that are mainly driven by sensory inputs or alternatively by anticipation. That is, while the proportion of HIT trials (≒55%) that is the same as the total FA rate (≒25%) may be attributed to amplified anticipatory processing, the remaining HIT trials (≒30% out of 55%) is likely to reflect sensory inputs for the target faces instead. Thus, FA trials are more likely to reflect top-down-related processing than are HIT trials. Here, trials are binary divided into higher and lower anticipation trials for simplicity, and the actual proportions may vary across participants. Download Figure 1-1, PNG file.

We used ultra-high-field 7 tesla (7T) fMRI with a spatial resolution of 0.8 mm to assess the activity of the pulvinar as well as V1 during the detection tasks. Such high spatial resolution enabled us to infer the cortical depth dependent activity of V1 (Norris and Polimeni, 2019). The cortical depth measurement is advantageous because the activity related to threat anticipation may be particularly observed in V1 superficial cortical depth, where non-sensory-driven activity has been observed (Muckli et al., 2015). We predicted that the anticipation-driven false perception of a fearful face may be accompanied with enhanced activity in the pulvinar and V1, as well as the potentially enhanced functional connectivity between these two areas.

## Materials and Methods

### Participants

We enrolled 12 participants (six males, mean age 23.7 ± SD 3.6, 2 left-handed), who provided written consent and received monetary reward after the experiment. Participants were all healthy and had normal or corrected-to-normal vision. The experiment protocol was approved by the ethical committee of Maastricht University. The data of one participant were removed from analysis due to excessive head motion (mean across runs > 4 mm).

We estimated that a sample size of *N* = 11 would be satisfactory to detect a medium to large effect size (*f* = 0.30) with an α power of 0.05 and power of 80% (G*Power version 3.1.9.2) as estimated with our 3T fMRI pilot results for pulvinar activity. Although we initially aimed for *N* = 12 to be conservative, one participant was removed from analysis as described above, leaving us with *N* = 11. Note that the sample size here is equivalent or larger than the related recent studies with 7T fMRI (e.g., four or 10 analyzed participants; Muckli et al., 2015; Kok et al., 2016).

### Stimuli

Face images of six models (three males) displaying fearful, happy, and neutral expressions were taken from the NimStim face stimulus set (Tottenham et al., 2009). We only included face images with the mouth open so that the local feature of an opened mouth alone would not enable detection of a fearful or happy rather than a neutral face. The images were gray-scaled and cropped into oval shapes to eliminate hair. They were then matched for luminosity, contrast, and spatial frequency spectrum with the SHINE toolbox (Willenbockel et al., 2010) implemented in MATLAB (R2011b, MathWorks). We refrained from further manipulation of stimulus properties, as excessive manipulation itself could unintentionally induce differential activity in V1, which is sensitive to lower-level stimulus properties.

### Experimental design

Participants completed two sessions, in which either fearful or happy face targets were presented in a counterbalanced order.

The fearful face detection task required participants to detect a briefly presented fearful face followed by a mask (neutral face; Fig. 1). On each trial, either a fearful or neutral face appeared as a target for 33 ms. Following a blank of 17 ms, a neutral face was presented for 133 ms as a mask that rendered the target face less visible but did not completely abolish its visibility. The model for the mask face was always different from the model for the target face. We used a neutral face as a mask instead of simpler images such as checkerboards, because V1 typically shows preferential activity toward simpler visual features (e.g., contrast and edges) and use of simple image masks could have interfered with the measurement of the critical activity in V1 related to face processing.

To further control task difficulty, the contrast of the target face was reduced to 35% of the contrast of the mask face, as determined during our pilot study. After 7.5 s from the target onset, response key assignment was shown on the screen. A fearful or neutral face response was assigned randomly to either the left or right key. Participants were instructed to respond with their right hand their first guess on whether they had perceived a fearful or neutral face target within a 2-s time window. After a jittered intertrial interval from the offset of response time window (8, 10.5, or 13 s), another trial was initiated. There were 24 trials in each of eight runs (8 min 10 s per run), comprising 12 trials each for fearful and neutral face targets. There was no feedback provided on each trial. The order of trials was randomized.

In the control task session, we used happy faces, which are emotionally salient but non-threatening. The task was otherwise identical to the fear condition with the same neutral face stimuli. The fearful and happy face sessions were conducted on two separate days in a counterbalanced order across participants. Stimuli were presented with Psychtoolbox (Brainard, 1997) implemented in MATLAB (R2012a, MathWorks).

For the data analysis the percept of participants was classified as follows: The correct percept of the presented fearful or happy target face was classified as HIT, whereas the false percept of a fearful or happy face in trials where a neutral face was presented was classified as FA. The correct percept of the presented neutral face was classified as correct rejection (CR), whereas the incorrect percept of a neutral face in trials where a fearful or happy face was presented was classified as MISS.

### fMRI data acquisition

MRI data were acquired with a 7T Magnetom scanner (Siemens) at the Scannexus facility located at the Department of Cognitive Neuroscience, Faculty of Psychology and Neurosciences, Maastricht University, with a Nova 1-transmitter/32-receiver head coil (Nova Medical). For functional data acquisition, 2D gradient-echoplanar images (EPI) were acquired at 0.8-mm isotropic resolution, with the following parameters: repetition time (TR) = 2500 ms, echo time (TE) = 21.8 ms, flip angle = 80°, GRAPPA acceleration factor = 3, matrix size = 154 × 236, field of view (FOV) = 123 mm × 188 mm, slice thickness = 0.8 mm, number of slices = 40, no gaps, echo spacing = 1.04 ms, bandwidth = 1116 Hz/Px, no multi-band acceleration. The slices were oriented to cover both the pulvinar and V1. To achieve maximal brain coverage with these parameters, right to left (RL) phase encoding was used for the task runs, so that the temporal areas outside the FOV were folded within the FOV and were trimmed later offline. A run of five TRs with the same parameters but with the opposing left to right (LR) phase encoding direction was acquired immediately before each task run for offline top-up EPI distortion correction (for more details, see below, fMRI processing). A separate run (3 min 30 s) was acquired to define V1 (see below, Retinotopic delineation of V1).

For anatomic data in nine participants, a T1-weighted scan and a proton-density-weighted scan were acquired with a resolution of 0.6 mm isotropic (FOV = 229 mm × 229 mm, matrix size = 384 × 384, flip angle = 5. T1-weighted: TR = 3100 ms, TE = 2.52 ms; proton-density-weighted: TR = 1440 ms, TE = 2.52 ms). For the other three participants, we used anatomic images with a spatial resolution of 0.7 mm isotropic from previous unrelated experiments.

### fMRI processing

fMRI analyses were conducted in BrainVoyager 20.2 (Brain Innovation). For preprocessing of fMRI data, we trimmed the lateral sides of EPI images by a small amount (60 voxels) to remove the folded-in tissue outside the FOV. The folding-in and trimming did not affect the coverage of the bilateral pulvinar and V1. The trimmed EPI images were then slice time corrected (sinc interpolation) and corrected for 3D rigid body motion (trilinear/sinc interpolation). Distortions of the EPI images from the task runs were adjusted against EPI images taken immediately before each task run with the opposing encoding phase (Andersson et al., 2003), with the BrainVoyager plugin COPE (https://support.brainvoyager.com/brainvoyager/available-tools/86-available-plugins/62-epi-distortion-correction-cope-plugin). EPI images then underwent temporal high-pass filtering with 2 cycles per run. That is, signals with temporal frequency with the cycle smaller than half the length of a run (i.e., 245 s) were removed with discrete Fourier filter.

After the preprocessing EPI images were manually aligned to the anatomic images in BrainVoyager including optimization of alignment around the posterior portion of the brain encompassing V1 and the pulvinar. From the total of runs those with 3D motion larger than 2.5 mm were discarded from analyses, because large motion induced excessive and/or unique EPI distortion that interfered with precise alignment and the subsequent cortical depth-specific analyses. One participant’s data were excluded from further analysis due to excessive head movements (>4 mm). For the remaining participants, the run numbers included in analyses did not differ between the fearful face task and the happy face task (mean ± SD = 7.64 ± 0.67, 7.45 ± 0.82, respectively; *t*_(10)_ = 0.48, *p* = 0.640). The mean number of trials entered into the fMRI analyses for each participant, after excluding runs with excessive head motion was: fearful HIT, mean ± SE = 45.64 ± 4.00; fearful FA, 26.64 ± 3.48; happy HIT, 54.18 ± 4.32; happy FA, 21.18 ± 2.93.

Task-related activity was then estimated with a deconvolution analysis, in which responses for successive five points (2.5 s × 5 TRs) were estimated, starting from the onset of the target face for each of the eight trial types (fearful face HIT, FA, CR, MISS, as well as happy face HIT, FA, CR, MISS). We used the deconvolution analysis because it does not assume a fixed hemodynamic response function, which is generally built based on the response properties in sensory cortical areas (Boynton et al., 1996; Glover, 1999) and is thus potentially less favorable for thalamic areas. The entire deconvolved time course in V1 and the pulvinar are shown in Extended Data Figure 2-1. Additionally, the GLM model included 6 head-motion nuisance parameters (three translation directions and three rotation axes). EPIs were spatially smoothed only when localizing the task-relevant voxels but were not smoothed when estimating the task-related activity to maintain laminar specificity.

**Figure 2.**
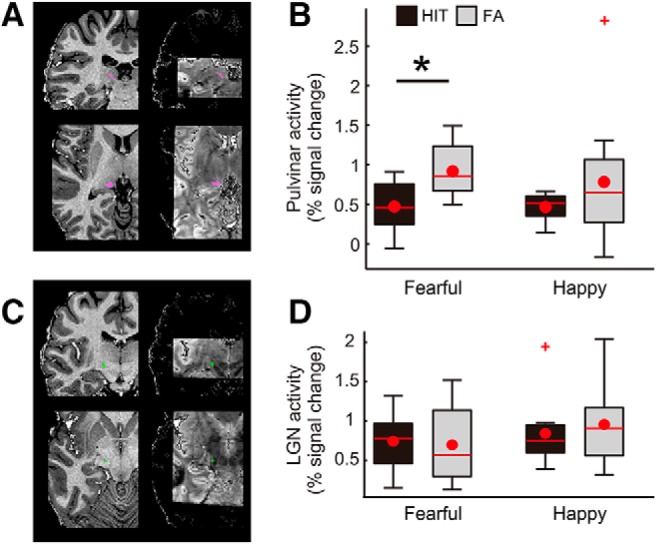
Activity in the pulvinar and LGN. ***A***, Demonstrations of pulvinar ROI from a representative participant, shown on the anatomic image (left panels) and EPI (right panels). ***B***, Pulvinar showed enhanced activity in FA trials relative to HIT trials during the fearful face detection task (*t*_(10)_ = –2.94, *p* = 0.015) but not during the happy face detection task (*t*_(10)_ = –1.23, *p* = 0.247). There was no significant interaction between percept type and emotion (*F*_(1,10)_ = 0.16, *p* = 0.702). ***C***, Demonstrations of the LGN ROI from an example participant. ***D***, Unlike the pulvinar, the LGN showed no differential activity between the percept types and facial emotions. Box plot shows upper (75%) and lower (25%) quartiles with median (red line) and mean (red dot), with whisker showing maximum and minimum value. An outlier (outside of ±2.7 SDs within a distribution for a given condition) is shown with a red cross; *p < 0.05. See also Extended Data Figures 2-1*A*, 2-2.

**Figure 3.**
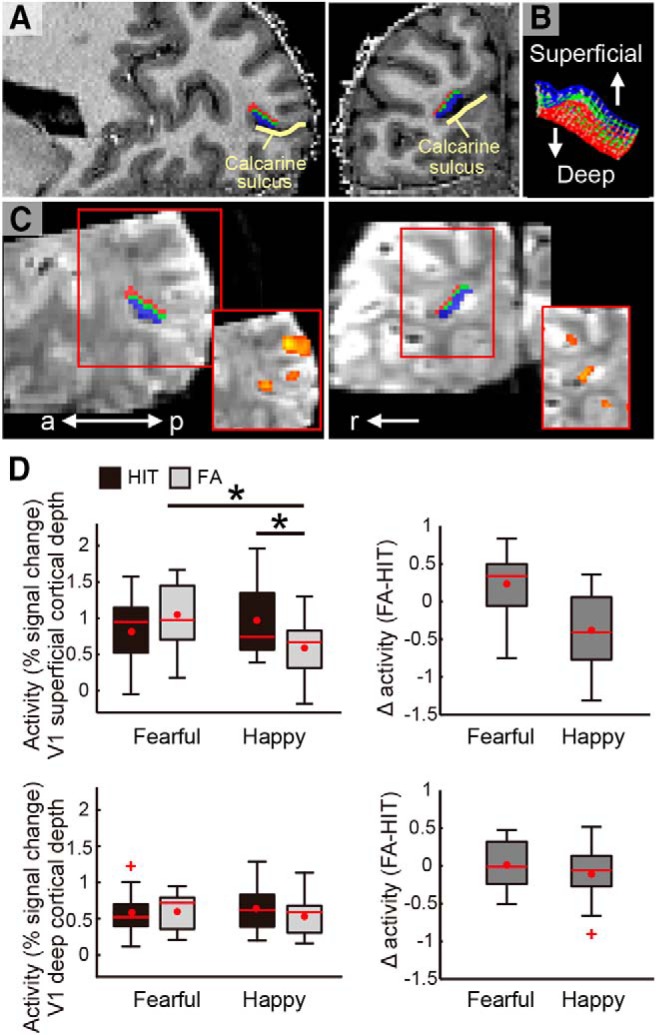
Demonstrations of V1 cortical depths and cortical depth dependent activity during the tasks. ***A***, V1 cortical depth is visualized on an anatomic image of a representative participant with sagittal (left panel) and coronal views (right panel). The voxels allocated to superficial (outwards to pial surface) and deep (inwards to white matter) cortical depths are shown in blue and red, respectively. The voxels allocated to the intermediate depth (shown in green) were disregarded in the main analyses (see Materials and Methods). ***B***, The cortical grid mesh within which the voxels were allocated. ***C***, The V1 cortical depth is visualized on EPI images (visualized in 3D for demonstrative purpose) in sagittal and coronal views (left and right, respectively). Red squares at the lower right demonstrate activity for all face targets relative to baseline on the EPI smoothed with a 3D kernel of 2.4-mm FWHM. ***D***, Peak activity at V1 superficial and deep cortical depths (upper and lower rows, respectively) in HIT and FA trials in the fearful face and happy face detection tasks. The difference in activity between FA and HIT trials are demonstrated in the right panel for each task, with a larger value indicating greater activity for FA than for HIT trials. Box plot shows upper (75%) and lower (25%) quartiles with median (red line) and mean (red dot), with whisker showing maximum and minimum value. An outlier (outside of ±2.7 SDs within a distribution for a given condition) is shown with a red cross. a: anterior, p: posterior, r: right; *p < 0.05. See also Extended Data Figures 2-1*B*, 3-1, 3-2, 3-3, 3-4.

10.1523/ENEURO.0429-19.2019.f2-1 Extended Data Figure 2-1 Pulvinar and V1 activity. ***A***, Deconvolved time course for the pulvinar for HIT and FA trials during the detection tasks with fearful and happy face targets (left and right panels, respectively). Error bar indicates standard error of mean. ***B***, Deconvolved time course of V1 for the HIT and FA trials during the fearful face detection task and happy face detection task (left and right panels, respectively). Error bar indicates standard error of mean. Related to Figures 2, 3. Download Figure 2-1, TIF file.

10.1523/ENEURO.0429-19.2019.f2-2 Extended Data Figure 2-2 Activity of the pulvinar on FA and HIT trials in each of its subregions. ***A***, The subregions composing the lateral, inferior, and medial portions of the pulvinar were defined based on a histological atlas (Chakravarty MM et al. 2006). The atlas was imported to BrainVoyager, and the entire pulvinar including all subregions was manually aligned to the pulvinar in each participant’s Native space. We included the subregions that compose the lateral, inferior, and medial portions of pulvinar which are widely implicated in visual processing (Pessoa and Adolphs 2010; Bridge et al. 2016), namely the nucleus pulvinaris oromedialis (lateral), nucleus pulvinaris orolateralis (lateral), pulvinar laterale (lateral), nucleus pulvinaris intergeniculatus (inferior), nucleus pulvinaris (medial), and pulvinar mediale (medial). The entire voxels within each subregion were used to estimate the activity level. ***B***, Similarly to the results in the main text (Fig. 2) with the functionally defined pulvinar ROIs, there was significantly larger activity on the FA trials of a fearful face relative to the FA trials of a happy face in two subregions, namely the nucleus pulvinaris oromedialis (lateral) and nucleus pulvinaris (medial). However, there was no significant interaction between emotion (fearful/happy) and condition (HIT/FA) in these two subregions (nucleus pulvinaris oromedialis: *F*_(1,10)_ = 4.496, *p* = 0.060; nucleus pulvinaris: *F*_(1,10)_ = 2.327, *p* = 0.158). The results suggested that one lateral and one medial subregion showed significantly larger activity on FA trials of a fearful face relative to FA trials of a happy face. Given that the same neutral faces were presented on these trials, the difference in activity is likely to be due to the perceived emotion driven by anticipation of a fearful versus happy face target. These results hint at the possibility that task-driven anticipation of threat signals may be coded in the medial pulvinar through its interaction with the prefrontal areas where task-set is generally represented (Lau and Passingham, 2007), while the lateral pulvinar may directly modulate V1 based on anticipation. However, these results should be treated as indicative as the results within these subregions were limited in that there was no significant interaction between conditions (HIT/FA) and emotions (fearful/happy) and that the relatively lower SNR in the inner brain area like the pulvinar may have interfered with the differentiation of subregion activities (see Materials and Methods, Estimation of tSNRs). Box plot shows upper (75%) and lower (25%) quartiles with median (red line) and mean (red dot), with whisker showing maximum and minimum value. An outlier (outside of ±2.7 SDs within a distribution for a given condition) is shown with a red cross; *p < 0.05 in planned *post hoc* tests. r: right, l: left, a: anterior, p: posterior. Related to Figure 2. Download Figure 2-2, TIF file.

10.1523/ENEURO.0429-19.2019.f3-1 Extended Data Figure 3-1 V1 activity in each participant at superficial (top row) and deep cortical depths (bottom row). For each subject, deconvolved time course of V1 during the HIT and FA trials during the fearful face detection task and happy face detection task (left and right panels, respectively) are shown. Subjects who showed numerically larger activity on FA trials of a fearful face relative to FA trials of a happy face, which is in line with the pattern of group level result shown in Figure 3C, are highlighted with red fonts (eight out of 11 participants). Related to Figure 3. Download Figure 3-1, TIF file.

10.1523/ENEURO.0429-19.2019.f3-2 Extended Data Figure 3-2 The results of gPPI analyses examining the connectivity between the pulvinar and V1. gPPI analyses were separately conducted, with the V1 superficial or deep cortical depth voxels as the seed ROI [see Materials and Methods, Generalized form of context-dependent psychophysiological interaction analysis (gPPI)]. Mean *t* values for the parameter estimates in gPPI for each trial type and seed ROI are shown. Only during FA trials with fearful faces, a significant modulation of connectivity was present between the pulvinar and V1 superficial layers (*t*_(10)_ = 3.981, *p* = 0.0026, one-sample *t* test against 0, significant after Bonferroni correction). We note that these results are only indicative, as there was no significant interaction between conditions (HIT/FA), facial emotion (fearful/happy), and V1 cortical depth (superficial/deep; *F*_(1,10)_ = 0.198, *p* = 0.666). There was no evidence that the connectivity between the pulvinar and V1 was modulated during other conditions in either V1 cortical depths. With the V1 superficial cortical depth as a seed ROI, fearful HIT: *t*_(10)_ = 0.403, *p* = 0.695, CI [-0.683, 0.985]; fearful FA: *t*_(10)_ = 3.981, *p* = 0.0026, CI [0.319, 1.128]; happy HIT: *t*_(10)_ = 1.869, *p* = 0.091, CI [–0.105, 1.120]; happy FA: *t*_(10)_ = 1.4364, *p* = 0.181, CI [–0.271, 1.253]. With the V1 deep cortical depth as a seed ROI, fearful HIT: *t*_(10)_ = 0.398, *p* = 0.699, CI [–0.641, 0.92]; fearful FA: *t*_(10)_ = 2.223, *p* = 0.05, CI [–0.001, 1.199]; happy HIT: *t*_(10)_ = 1.594, *p* = 0.142, CI [–0.15, 0.01]; happy FA: *t*_(10)_ = 1.152, *p* = 0.276, CI [–0.459, 1.441]. We note that the null results do not necessarily indicate the absence of effects, because a PPI analysis is generally low in power (O'Reilly et al., 2012) and tSNR in the pulvinar was relatively low in the current study. Future studies may further examine the potential changes in the functional connectivity with the pulvinar across the V1 cortical depths and experimental conditions. pulv: pulvinar, a: anterior, p: posterior. Download Figure 3-2, TIF file.

10.1523/ENEURO.0429-19.2019.f3-3 Extended Data Figure 3-3 Prior to the onsets of target faces, there was already enhanced activity in the pulvinar preceding the FA trial of a fearful face compared with a happy face (left panel). To estimate activity prior to the face onsets, the *z*-normalized signal change (%) in the preprocessed raw time course (see Materials and Methods, fMRI processing) was averaged between the two time points immediately before the face onsets (i.e., –5 and –2.5 s) relative to the preceding baseline (averaged between the two earlier time points, i.e., –10 and –7.5 s). A repeated-measures ANOVA with two factors of percept type (pre-HIT/pre-FA) and emotion (fearful/happy) revealed a significant interaction (*F*_(1,10)_ = 10.094, *p* = 0.010). *Post hoc* analyses revealed that pulvinar activity was significantly greater on FA trials of fearful faces compared with happy faces in the pre-onset period (*t*_(10)_ = 3.392, *p* = 0.007, *d* = 1.1). During the same pre-onset period, while pulvinar activity was significantly larger on HIT than on FA trials of happy faces (*t*_(10)_ = 2.386, *p* = 0.038, *d* = 0.7), there was a non-significant opposite trend such that activity was relatively greater on FA than on HIT trials of fearful faces (*t*_(10)_ = –2.147, *p* = 0.057, n.s.). Meanwhile, in the same pre-onset period, there was not yet any differential activity in the V1 superficial cortical depth (right panel), with no significant main effects or interaction between percept type and emotion (*ps* > 0.50, n.s.). These results suggest that anticipatory activity in the pulvinar modulated the response of the V1 superficial cortical depth triggered by the subsequent onset of a face target shown in Figure 3D. Box plot shows upper (75%) and lower (25%) quartiles with median (red line) and mean (red dot), with whisker showing maximum and minimum value; **p < 0.01, * *p* < 0.05, +*p* < 0.10. Related to Figures 2, 3. Download Figure 3-3, TIF file.

**Figure 4.**
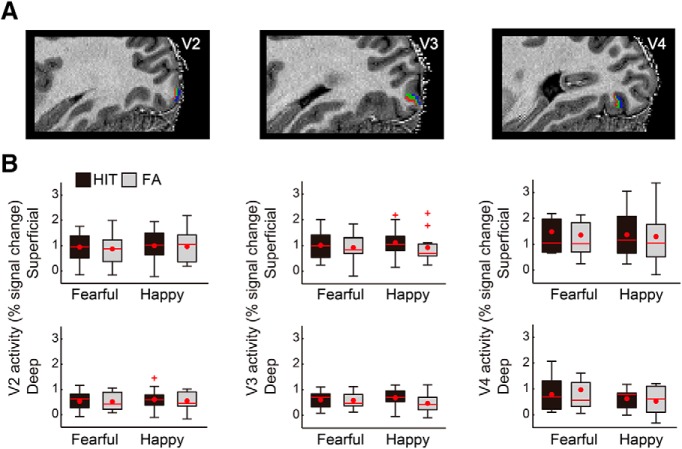
Control analyses showing no differential activity in V2, V3, and V4 in HIT compared with FA trials. ***A***, V2, V3, and V4 cortical depths are visualized on the anatomic image of an example participant, with the same color coding as for V1 shown in Figure 3. ***B***, Unlike V1, V2–V4 did not show any differential activity between the percept types and facial emotions, regardless of cortical depth. Box plot shows upper (75%) and lower (25%) quartiles with median (red line) and mean (red dot), with whisker showing maximum and minimum value. An outlier (outside of ±2.7 SDs within a distribution for a given condition) is shown with a red cross.

10.1523/ENEURO.0429-19.2019.f3-4 Extended Data Figure 3-4 The results of a control analysis showing no differential activity in the pulvinar and V1 on MISS versus CR trials. We examined whether similar results on FA trials of fearful faces (Figs. 3B, 4D) may be present on MISS trials, where a fearful face was presented but was not detected. While the results for FA trials of fearful faces may reflect enhanced top-down processing, other non-mutually exclusive possibilities are worth considering. Specifically, the results for FA trials may reflect a mere mismatch between sensory input and reported percept. Although this possibility is unlikely considering the fact that such results did not generalize to FA trials of happy faces, there remains a possibility that such input-to-percept mismatch may evoke certain neural activity only in anticipation of threat cues. We therefore examined whether the patterns of results we obtained from the contrast between FA and HIT trials may be also obtained from the contrast between MISS and CR trials, where neutral faces were perceived with or without mismatched sensory input, respectively. ***A***, Unlike the contrast of pulvinar activity between the HIT and FA trials shown in Figure 2, there was no difference in pulvinar activity between the MISS and CR trials regardless of the facial emotion [a non-significant interaction between percept type (MISS/CR) and emotion: F(1,10) = 0.013, p = 0.912]. Although we saw a trend for pulvinar activity to be larger for the fearful face detection task than for the happy face detection task, there was no significant main effect of emotion (F(1,10) = 2.365, p = 0.155). B, Similarly, unlike the contrast of V1 activity between the HIT and FA trials shown in Figure 3, there was no differential activity in V1 between the MISS and CR trials, regardless of emotion and cortical depth (a non-significant second-order interaction: *F*_(1,10)_ = 1.251, *p* = 0.290). While there was generally greater activity on MISS trials relative to CR trials that was not specific to any facial emotion and potentially reflected sensory inputs of salient emotional faces albeit undetected, there was no significant main effect of percept type (*F*_(1,10)_ = 2.573, *p* = 0.140). Box plot shows upper (75%) and lower (25%) quartiles with median (red line) and mean (red dot), with whisker showing maximum and minimum value. An outlier (outside of ±2.7 SDs within a distribution for a given condition) is shown with a red cross. Related to Figures 2B, 3D. Download Figure 3-4, TIF file.

### Localization of the pulvinar and LGN

We localized the pulvinar as the target region, as well as the LGN as a control region (Fig. 2). Each of the thalamic regions (the pulvinar and LGN) was first anatomically defined. Specifically, the pulvinar was located in the dorsal thalamus that is superior and medial to the LGN, located adjacent to the third ventricle (Kastner et al., 2004; Sprenger et al., 2012; Mai et al., 2015). To localize the pulvinar, we referred to the histologic atlas (Chakravarty et al., 2006) superimposed on the high-resolution T1 resampled at 0.8 mm in native space. To specifically select task-relevant voxels within the anatomically localized pulvinar, functional images from the task runs were smoothed with a Gaussian kernel of 2.4-mm full width at half maximum (FWHM). Task-relevant voxels were then defined based on activation at the temporal peak of the time course (5 s from the target face onset while considering a hemodynamic delay of 5 s, i.e., 2 TRs × 2.5 s) that was larger than baseline at a threshold of *p* < 0.01 uncorrected to compensate for a generally lower tSNR in subcortical areas (see below, Estimation of tSNRs). Note that task-relevant voxels were selected based on the target face onsets from all trials including all conditions, to minimize any bias toward one particular trial type over another.

Pulvinar regions of interest (ROIs) were located in both hemispheres for all participants except for one participant (ROI in the left hemisphere only). LGN ROIs were located in both hemispheres for five participants, while they were located in either the right or left hemisphere for four and two participants, respectively. Inability to locate the ROIs in both hemispheres in some participants is likely due to the generally hindered SNR toward deeper brain areas (see below, Estimation of tSNRs). For participants with pulvinar and/or LGN ROI(s) located in both hemispheres, activity was estimated for each hemisphere and averaged.

### Retinotopic delineation of V1

To delineate V1 in each participant, a retinotopy run was acquired. During the run, color/luminance-flickering wedge-shaped checkerboard patterns (30° in polar angle) were presented along the horizontal or vertical meridian alternately for 15 s, each with six repetitions, following procedure in Lafer-Sousa and Conway (2013). Checkerboard patterns were flickered at 4 Hz and were displayed in one of four color combinations (red/green, blue/yellow, black/white, and magenta/cyan) to activate neurons with various response profiles and enhance the signals to identify the boundaries between the cortical areas. The boundaries of V1 were delineated with a general linear model contrasting activity between the horizontal and vertical presentation periods as previously described (Lafer-Sousa and Conway, 2013). V2, V3, and V4 were delineated in a similar manner and served as control regions. The boundary of V4 was located while additionally referring to its predefined anatomic landmarks (Witthoft et al., 2014).

### Anatomic image processing and cortical depth-specific estimation of V1 activity

Inhomogeneity of T1-weighted images was corrected by dividing the original image intensities by the proton density images (Van de Moortele et al., 2009). Subsequently, the corrected T1 image was resampled at a resolution of 0.8 mm to match the resolution of EPI. The boundaries of gray-white matter and the pial surface were first estimated with BrainVoyager 20.2, and further corrected manually, to improve the precision and to remove the blood vessels and dura mater based on image intensity. The anatomic image was not transformed to standardized coordinates but was kept in native space, to reduce resampling and maintain its laminar properties undistorted.

As was done for the pulvinar, the task-relevant voxels were selected within the delineated V1 of each hemisphere. Specifically, the task-relevant voxels were defined based on the contrast between all target face onsets versus baseline at a threshold of *p* < 0.001 uncorrected, using the parameter estimate at the temporal peak of the time course at 5 s (two TRs × 2.5 s). The task-relevant voxels within V1 were successfully located in both hemispheres in 8 participants, while they were located in only one hemisphere in the remaining three participants (right only, *N* = 1; left only, *N* = 2). For participants with peaks located in both hemispheres, the estimates of activity were averaged between the hemispheres for each visual cortical area (e.g., V1).

To define the cortical depths of V1, we used the Laplace equation to estimate cortical thickness and then obtained an equidistant definition of depth with respect to the local thickness (Muckli et al., 2015; De Martino et al., 2018) at three depth levels (from 25%, 50%, and 75% depth levels relative to the local cortical depth in an inward direction) centering around the spatial activity peak (i.e., voxel with highest activation level) among the pre-defined task-relevant voxels with 15 × 15 grids of 0.5 voxels. Individual voxels were assigned to the adjacent cortical depth, and were used as ROIs in the subsequent analyses (Fig. 3). We confined our analyses to voxels allocated to the superficial and deep cortical depth groups, which roughly correspond to cortical layers 1–3 and layers 5–6, respectively, due to the difference in anatomic thickness of each layer (de Sousa et al., 2010; Kok et al., 2016). The task-relevant voxels assigned to the superficial and deep depths covered an average of 22.7% (SE = 4.0%) of the retinotopically delineated V1 (see above, Retinotopic delineation of V1). This relatively small coverage by the task-relevant voxels is likely to be due to lower contrast and luminosity as well as smaller stimulus size relative to the V1 delineation run (i.e., the visual angles spanned by the checkerboards wedges and the task face images were 10.4° × 10.4° and 6.1° × 4.5°, respectively). The cortical depth dependent activity was estimated from the unsmoothed functional data to maintain the original spatial resolution (0.8 mm).

We localized the control areas V2–V4 in a similar manner as V1. The task-relevant voxels within V2 and V3 were successfully located in both hemispheres in nine participants, while they were localized in only one hemisphere in 2 participants (V2 right only, *N* = 1; V2 left only, *N* = 1; V3 right only, *N* = 1; V3 left only, *N* = 1). The cortical depths of V2 and V3 were defined separately for the dorsal and ventral areas, and the voxels for each cortical depth were combined between the dorsal and ventral areas. The task-relevant voxels within V4 were located in both hemispheres in all participants.

### Estimation of tSNRs

The SNR of the time series (tSNRs) for the pulvinar, LGN, and V1 superficial and deep cortical depths were assessed with VTC inspector plugin in BrainVoyager (Brain Innovation). tSNR was assessed for each ROI from the time course in initial runs of the tasks and averaged across participants. The mean tSNR (±SD) was 8.12 (±1.03) for the pulvinar, 7.06 (±0.38) for LGN, 18.15 (±4.48) for V1 superficial cortical depth and 18.04 (±3.92) for V1 deep cortical depth. In line with the previous report that the gradient-echo imaging sequence employed here yields higher SNR toward the surface of the cortex (De Martino et al., 2018), thalamic areas (i.e., the pulvinar and LGN) that were deeper inside the brain and further from the coil had lower tSNR relative to V1 on the outer brain.

### Generalized form of context-dependent psychophysiological interaction analysis (gPPI)

To examine whether functional connectivity between the pulvinar and V1 was enhanced during certain trial types (e.g., FA trials with fearful faces), we conducted a pair of gPPI analyses (McLaren et al., 2012): one with V1 superficial cortical depth voxels as the seed ROI, and the other with V1 deep cortical depth voxels as the seed ROI. For each analysis, the GLM model included regressors for each of the eight trial types (i.e., HIT, FA, CR, MISS for fearful and happy faces) convolved with the canonical two-γ HRF, a regressor for the z-normalized time course of the seed ROI, regressors for PPI terms (i.e., seed time course × trial type regressor), six nuisance regressors of 3D head motions (three translation directions and three rotation axes). The GLM analysis was run for each participant, and the parameter estimates (β values) were extracted within the pulvinar ROI for the PPI term of HIT and FA trials of fearful and happy faces (i.e., the four critical trial types included in the main results). In the group-level analysis, *t* values for the parameter estimates for each trial type and seed ROI in gPPI (Extended Data Fig. 3-2) were tested against 0 with a one-sample *t* test, with Bonferroni correction for eight conditions (i.e., *α* = 0.05/8, HIT/FA × fearful/happy × V1 superficial/deep cortical depths).

### Statistical analysis

We conducted subject-level analyses of fMRI data in BrainVoyager 20.2 (Brain Innovation; see above, fMRI processing), and subsequently, conducted group-level repeated-measures ANOVAs in IBM SPSS Statistics (version 18). Behavioral performance was also analyzed in IBM SPSS.

Following previous studies (Keselman and Keselman, 1993; Tamaki et al., 2016), when an omnibus three-way repeated-measures ANOVA revealed a significant three-way interaction, we conducted two-way repeated-measures ANOVAs to locate a simple interaction effect. Detection of a significant two-way interaction was followed by *t* tests (two-tailed) to examine simple main effects. Similarly, when an omnibus repeated-measures ANOVA initially involved only two factors (i.e., two-way), detection of a significant interaction was followed by *t* tests (two-tailed). The series of *t* tests were not susceptible to the inflation of type 1 error as they followed significant interactions in the initial omnibus ANOVAs, as has been validated and commonly practiced previously (Keselman and Keselman, 1993; Cohen, 2004; Tamaki et al., 2016). All *t* tests reported in this article were two-tailed.

**Table 1.** Statistical table

	Data structure	Type of test	Confidence interval
a	Normal distribution	Paired-sample *t* test (two-tailed)	[–0.009, 0.097]
b	Normal distribution	Paired-sample *t* test (two-tailed)	[–0.172, –0.052]
c	Normal distribution	*Post hoc t* test following ANOVA (two-tailed)	[–0.782, –0.108]
d	Normal distribution	*Post hoc t* test following ANOVA (two-tailed)	[–0.888, 0.256]
e	Normal distribution	*Post hoc t* test following ANOVA (two-tailed)	[–0.391, 0.661]
f	Normal distribution	*Post hoc t* test following ANOVA (two-tailed)	Fearful [–0.335, 0.424]; happy [–0.500, 0.272]
g	Normal distribution	*Post hoc t* test following ANOVA (two-tailed)	[0.025, 0.735]
h	Normal distribution	*Post hoc t* test following ANOVA (two-tailed)	[–0.542, 0.071]
i	Normal distribution	*Post hoc t* test following ANOVA (two-tailed)	[–0.871, –0.048]
j	Normal distribution	One-sample *t* test (two-tailed)	[0.319, 1.128]

## Results

### Behavioral performance

Perception of a fearful face reported on presentation of a fearful face target was classified as HIT, whereas perception of a fearful face reported on presentation of a neutral face was classified as a FA and similarly for the happy versus neutral faces in the control task. The ratio of FA was similar between the fearful face and the happy face detection task (28.5 ± 3.5% and 24.1 ± 3.1%, respectively), and the rates in each task did not significantly differ from each other (*t*_(10)_ = 1.85, *p* = 0.095^a^, Table 1). The ratio of HIT trials was higher for the happy face (mean ± SE = 60.4 ± 4.4%) than for the fearful face detection task (49.2 ± 4.4%; *t*_(10)_ = –4.16, *p* = 0.002^b^). This is consistent with previous literature showing that explicit labeling of happy faces is easier than that of negative faces (Calvo and Lundqvist, 2008). Our choice of facial images with open mouth may have additionally contributed to relatively poorer detection of a fearful face against a neutral face target or mask because a neutral face may look more similar to a fearful face with opened mouth.

### Pulvinar activity during the detection tasks

Our primary interest was how activity of the higher-order thalamic area pulvinar (Fig. 2*A*) may differ between HIT and FA trials, and whether such a difference is specific to the fearful face detection task.

We observed that pulvinar activity was greater on FA trials than on HIT trials (Fig. 2*B*), as demonstrated by a main effect of percept type in a repeated-measures ANOVA (*F*_(1,10)_ = 8.31, *p* = 0.016). Greater activity in FA trials relative to HIT trials was observed during the fearful face detection task (*t*_(10)_ = –2.94, *p* = 0.015^c^) but not during the happy face detection task (*t*_(10)_ = –1.23, *p* = 0.247^d^). There was no significant difference between FA trials of the fearful face detection task and that of the happy face detection tasks (*t*_(10)_ = 0.57, *p* = 0.580^e^). There was no significant interaction between percept type and emotion (*F*_(1,10)_ = 0.16, *p* = 0.702). The main effect of percept type independent of emotion suggests that the pulvinar shows enhanced activity with a false percept in general, as has been reported in a previous study during a false detection of change in non-emotional stimuli (Pessoa and Ungerleider, 2004; for the deconvolved time course of the pulvinar, see also Extended Data Fig. 2-1*A*).

As a control analysis for the pulvinar activity, we examined the activity of a first-order thalamic region, the LGN (Fig. 2*C*). Our analysis showed that the activity level of the LGN was not altered in FA trials of fearful faces (Fig. 2*D*). Neither the main effect of percept type nor that of emotion was significant in a repeated-measures ANOVA (*F*_(1,10)_ = 0.10, *p* = 0.759; *F*_(1,10)_ = 3.01, *p* = 0.113, respectively^f^). The two-way interaction was also non-significant (*F*_(1,10)_ = 0.36, *p* = 0.560). The result of this control analysis suggests that the pulvinar, rather than the thalamic areas in general, contributed to FA trials.

### Cortical depth-dependent V1 activity during fearful face perception

While pulvinar activity alone did not dissociate between fearful and happy faces (Fig. 2), we observed that V1 superficial activity did (Fig. 3*D*). A repeated-measures ANOVA revealed a second-order interaction between percept type (HIT/FA), emotion (fearful/happy), and cortical depth (superficial/deep) of V1 (*F*_(1,10)_ = 5.67, *p* = 0.039). This interaction was due to the fact that percept type and emotion interactively modulated V1 activity at superficial cortical depths (*F*_(1,10)_ = 7.84, *p* = 0.019), but not at deep cortical depths (*F*_(1,10)_ = 0.67, *p* = 0.431; see Extended Data Fig. 2-1*B* for the deconvolved V1 time course and Extended Data Fig. 3-1 for the results separately plotted for individual participants).

At the superficial depth, V1 activity in FA trials of a happy face was less than that in HIT trials of a happy face (*t*_(10)_ = 2.39, *p* = 0.038^g^). This result is consistent with a previous study demonstrating that V1 activity levels are typically greater for HIT than for FA trials in a visual detection task with non-threatening targets (Ress and Heeger, 2003), although cortical depth dependent activities were not reported.

Contrary to the happy face detection task, V1 superficial depth activity in FA trials during the fearful face detection task was similar to or even numerically larger than that in HIT trials (*t*_(10)_ = –1.71, *p* = 0.117^h^). Indeed, *post hoc* analysis showed that V1 superficial depth activity in FA trials of a fearful face was significantly greater than that in FA trials of a happy face (*t*_(10)_ = –2.49, *p* = 0.032^i^), although the same neutral faces were presented with the only difference being the task context to anticipate either a fearful or happy face target. These results are consistent with the idea that anticipation-driven activity, which is more evident in the FA trials than in the HIT trials, was added on top of the smaller input-driven activity in FA trials. In other words, our results suggest that V1 superficial depth activity may reflect excessive top-down processing in anticipation of fearful face targets, which does not generalize to mere anticipation of any emotionally salient target (i.e., happy faces).

### Connectivity between the pulvinar and V1 during fearful face perception

The aforementioned results showed that V1 superficial depth activity in FA trials was enhanced only for fearful faces and pulvinar activity was higher in FA trials than in HIT trials regardless of facial emotion. Given that there is a reciprocal interaction between the pulvinar and V1 including modulatory input from the pulvinar to V1 superficial layers (Shipp, 2003, 2016; Purushothaman et al., 2012; Cicmil and Krug, 2015; Bridge et al., 2016; Roth et al., 2016), it is possible that the enhanced V1 superficial depth activity in FA trials of fearful faces is at least partly related to its communication with the pulvinar. We examined whether a modulation of activity was present between the pulvinar, and the superficial or deep V1 voxels using gPPI (McLaren et al., 2012). A functional coupling was present between the pulvinar and V1 superficial depth in FA trials for fearful faces (*t*_(10)_ = 3.98, *p* = 0.003, one sample *t* test against 0^j^; Extended Data Fig. 3-2), but not in the other trial types (fearful HIT: *t*_(10)_ = 0.40, *p* = 0.695; happy HIT: *t*_(10)_ = 1.87, *p* = 0.091; happy FA: *t*_(10)_ = 1.44, *p* = 0.181). In addition, another analysis hinted that there was already enhanced activity in the pulvinar before the onset of FA trials with fearful faces but not in V1 superficial depth (Extended Data Fig. 3-3). This result, together with the result of gPPI analysis, suggests that the modulatory input from the pulvinar may have contributed to the enhanced V1 superficial activity in FA trials with fearful face percept, although future studies need to directly test the specific direction of interaction between the two regions. There was no significant interaction with V1 cortical depth and facial emotions in the gPPI result (*F*_(1,10)_ = 0.20, *p* = 0.666; Extended Data Fig. 3-2).

### Control analyses

To examine the specificity of our results, we conducted a series of control analyses. We particularly examined the potential additional contributions of higher visual cortical areas, V2, V3, and V4 in the FA trials for fearful faces. The activity of these areas was examined separately for their superficial and deep cortical depths in a manner similar to V1 (Fig. 4*A*; Materials and Methods). Other higher visual areas such as fusiform areas were outside of the fMRI coverage.

Unlike V1, we found that the activity of V2–V4 was not enhanced in the FA trials of fearful faces (Fig. 4*B*). With V2, a second-order interaction between percept type, emotion, and cortical depth was non-significant (*F*_(1,10)_ = 0.40, *p* = 0.543). The main effects of percept type and emotion were also non-significant (*F*_(1,10)_ = 0.61, *p* = 0.452; *F*_(1,10)_ = 0.24, *p* = 0.635, respectively), while only the main effect of cortical depth was significant (*F*_(1,10)_ = 21.25, *p* = 0.001). Similarly, with V3, a second-order interaction was non-significant (*F*_(1,10)_ = 0.27, *p* = 0.618). Although there was a general trend that the activity on HIT trials was larger relative to FA trials, the main effect of percept type did not reach significance (*F*_(1,10)_ = 4.53, *p* = 0.059). The main effect of emotion was also non-significant (*F*_(1,10)_ = 0.02, *p* = 0.895), while a main effect of cortical depth was significant (*F*_(1,10)_ = 20.43, *p* = 0.001). Likewise, with V4, a second-order interaction (*F*_(1,10)_ = 1.03, *p* = 0.335) as well as the main effects of percept type and emotion were non-significant (*F*_(1,10)_ = 0.12, *p* = 0.732; *F*_(1,10)_ = 0.85, *p* = 0.370, respectively), while only the main effect of cortical depth was significant (*F*_(1,10)_ = 18.52, *p* = 0.002).

As an additional control analysis, we examined whether similar results to those observed in FA trials of fearful faces would be present in MISS trials, in which a fearful face was presented but not detected. While the aforementioned results for FA trials of fearful faces may reflect enhanced anticipatory processing as we speculated earlier, other non-mutually exclusive possibilities are worth considering. Specifically, the results in FA trials may reflect a mere incorrect response to a presented face image, in which case similar results as FA trials would be expected in MISS trials. Contrary to this possibility, we did not observe any notable results specific to MISS trials of fearful faces either in V1 or in the pulvinar (a non-significant interaction between percept type (MISS/CR) and emotion: *F*_(1,10)_ = 0.01, *p* = 0.912; Extended Data Fig. 3-4), excluding the possibility that the results in FA trials of fearful faces merely reflected a mere mismatch between the input and percept.

Taken together, the analyses suggest that the results of pulvinar and V1 superficial cortical depth in false perception of fearful faces were not mirrored in the LGN or in V2–V4. The findings also indicate that the results of pulvinar and V1 were not explained away either by mere anticipation of any emotionally salient target (i.e., happy face) or by mere mismatch between percept and input, suggesting the specificity of the involvement of pulvinar-to-V1 input in false perception of fearful faces.

## Discussion

We observed that the false percept of anticipated but not presented fearful face relative to that of a happy face was accompanied by increased activity in superficial cortical depth of V1, which constitutes the earliest stage of the visual cortical hierarchy. This enhanced V1 activity may particularly contribute to the anticipatory perception of threat signals, as it did not generalize to the perception of non-threatening signals, i.e., happy faces. Although preliminary, we additionally observed that the connectivity between the pulvinar and V1 superficial cortical depth was enhanced when participants falsely perceived fearful faces. These results suggest a potential role of the pulvinar and V1 in preparing the visual system to perceive an anticipated threat.

It may be counterintuitive to expect a crucial role of V1 in the perception of fearful faces, given that V1 is fine-tuned to low-level visual features that would only partially constitute the visual properties of fearful faces. Nevertheless, recent findings suggest that a lower visual cortical area can reflect higher-level features, when the prediction signals for such features originate from a higher area (Schwiedrzik and Freiwald, 2017). While such top-down signals are typically expected to descend from higher cortical areas, it has recently been speculated that the pulvinar also contributes to such top-down signals (Kanai et al., 2015), including contextual signals (Roth et al., 2016). Given that the pulvinar appears capable of coding threat signals, including a complex fearful face (de Gelder, 2006; Pessoa and Adolphs, 2010), one possibility is that the pulvinar serves as one of the critical regions to modulate the early stage processing in V1 rapidly biasing visual cortical processing toward threat perception.

In line with this, a recent MEG study with human participants demonstrated that the neural activity driven by the inputs of facial images is better explained by a dynamic causal model which considers the pulvinar-to-V1 input, and that such input is modulated by the presence of fearful expression in faces (McFadyen et al., 2017). While this study suggests the role of the pulvinar in input-driven relaying of information to V1, our study suggests that the pulvinar may play an additional role in modulating activity in the early stage of visual cortex in anticipation of threat-relevant signals in humans.

Interpretation about the direction of the interaction between the pulvinar and V1 in the current study has some limitations. First, the temporal resolution of fMRI was not high enough to detect the precedence of one region over the other, which may be compensated for in a future study using MEG. Secondly, the portions of the pulvinar active for the FA of fearful faces were not always symmetrical between the hemispheres and did not always converge to the anatomic subregions that have direct communication with V1 (Bridge et al., 2016; Extended Data Fig. 2-2). As the localization of the subregions of the pulvinar may be partly precluded because of relatively lower SNR (see Materials and Methods, Estimation of tSNRs), future studies may adopt alternative fMRI sequences for a better signal in the pulvinar. Future studies may also examine whether the current results generalize when facial images and masks were controlled differently (e.g., faces with closed mouth and non-facial masks).

The absence of enhanced V2–V4 activity in the FA trials of fearful faces may be surprising considering that the pulvinar and V1 could both drive V2 activity (Marion et al., 2013). However, these null results does not necessarily indicate that the enhanced activity in the pulvinar and/or V1 have no subsequent effect in higher visual areas V2–V4 in the FA trials of fearful faces. This is because the input-driven activity in V2–V4 is supposedly higher in HIT trials relative to FA trials (Ress and Heeger, 2003), especially due to salient sensory inputs of the fearful face targets. Thus, the absence of activity difference between the FA trials and HIT trials in these downstream areas of V1 may instead suggest that the anticipation-driven activity in the pulvinar and/or V1 may have compensated the originally lower activity in V2–V4 in the FA trials of fearful faces, making such activity comparable to the HIT trials of fearful faces.

Note also that these null results do not exclude that other areas besides the pulvinar and V1 were involved in false percept (i.e., FA trials) of fearful faces. Future studies should investigate whether and how the pulvinar and V1 may interact with other areas not covered in this study, such as the amygdala, fusiform face area and prefrontal areas. Whole brain analyses, which may help address such questions, were not performed because of the restricted brain coverage (see Materials and Methods, fMRI data acquisition), and because the spatial distortion and/or SNR were expected to be highly inhomogeneous even across the covered brain areas due to ultra-high magnetic field (see Materials and Methods, Estimation of tSNRs).

How false perception of non-threatening cues, such as happy faces, emerges remains to be investigated. One possibility is that such a percept would reflect top-down modulation of visual cortical areas higher than V1, similarly to the processing of facial identity (Schwiedrzik and Freiwald, 2017). Although the control analyses did not support the contribution of V2–V4 to the false perception of happy face targets, one possibility may be that visual areas higher than V4, not covered in this study (to allow for the spatial resolution desired), contributed here.

How the subjective experience of falsely perceiving an unpresented fearful face may have differed from that of correctly perceiving a presented fearful face is one remaining interesting question. One way to examine the potential difference could be to assess perceptual confidence. Another way could be to measure the whole brain activity to examine whether action related activity in motor areas and/or the pulvinar (Dominguez-Vargas et al., 2017) may have also contributed to bias responses in false perception, although it is noteworthy that response bias could be explained by the perceptual processing itself (Rahnev et al., 2011).

One potential limitation of the current method to estimate the cortical depth dependent activity is that the gradient-echo imaging sequence employed here is known to have a better SNR toward the surface of the cortex (De Martino et al., 2018). This SNR difference may mean that the study was underpowered to elucidate potential additional contributions of V1 at the deep cortical depth, although a previous study with similar 7T fMRI parameters has successfully elucidated significant effects in V1 even at its deep cortical depths (Kok et al., 2016) and the current study had similar tSNR between the cortical depths (see Estimation of tSNRs). The current results certainly suggest the involvement of V1 in the anticipatory processing of threat, but its layer-specificity may be further investigated in future studies with fMRI sequences other than gradient-echo.

The notion that the pulvinar plays a role in top-down modulation of fearful face processing is not mutually exclusive with the traditional view emphasizing the role of the pulvinar in the subcortical route (“low road”) to process the presented threat signals in an input-driven manner. The subcortical route is thought to bypass the cortex to rapidly relay the retinal input to the amygdala via the superior colliculus and pulvinar (LeDoux, 1996; de Gelder, 2006). Such coarse input-driven processing has been speculated to result in erroneous, false perception of threat signals (LeDoux, 1996). Although the function of such a subcortical route may be relatively degraded in humans compared with other species such as rodents (Pessoa and Adolphs, 2010; Bridge et al., 2016), its contribution has been demonstrated in humans (Tamietto and de Gelder, 2010; Tamietto et al., 2012; Méndez-Bértolo et al., 2016).

Overall, the current results suggest that anticipatory processing of threat signals is distinct from the input-driven processing of threat signals, with the former uniquely involving the engagement of the pulvinar and V1 which constitute the earliest stage of visual processing hierarchy. Such anticipatory processing of threat may contribute to the perception of threat-relevant images in the absence of corresponding sensory inputs as in clinical cases of flashbacks reported in post-traumatic stress disorder.

## Synthesis

Reviewing Editor: Carmen Sandi, Swiss Federal Institute of Technology Decisions are customarily a result of the Reviewing Editor and the peer reviewers coming together and discussing their recommendations until a consensus is reached. When revisions are invited, a fact-based synthesis statement explaining their decision and outlining what is needed to prepare a revision will be listed below. The following reviewer(s) agreed to reveal their identity: NONE.

This is a revised manuscript of a previously reviewed version. In this current version, the authors have faithfully and thoroughly integrated the reviewers recommendations and, as it stands now, the manuscript represents strong science advancing data on a timely topic. Using state-of-the-art behavioral and imaging methods (fMRI at 7T with a spatial resolution of 0.8 mm), the study addresses the neural engagement of anticipatory processing during the early stages of visual processing, in particular in the pulvinar and visual cortex V1. Importantly, the high spatial resolution achieved with the employed methodology allowed the authors to infer cortical-depth dependent activity of V1. Specifically, the study shows that the false (not present) percept of anticipated fearful (relative to happy) faces leads to increased activity in brain regions involved in earliest stages of visual cortical hierarchy (i.e., superficial cortical depth of V1), suggesting that this engagement is involved in anticipatory perception of threat signals. The authors also found preliminary evidence (that will require future confirmation) for enhanced connectivity between activity in the pulvinar and V1 superficial cortical depth when participants falsely perceived fearful faces and suggest a role for these brain regions in the preparation of the visual system to perceive anticipated threats.
