# Supplementary figures and images for: Threat Anticipation in Pulvinar and in Superficial Layers of Primary Visual Cortex (V1). Evidence from Layer-Specific Ultra-High Field 7T fMRI
Source: eNeuro. 2019 Dec 9;6(6):ENEURO.0429-19.2019. doi: 10.1523/ENEURO.0429-19.2019 (PMC6901684; doi:10.1523/ENEURO.0429-19.2019)

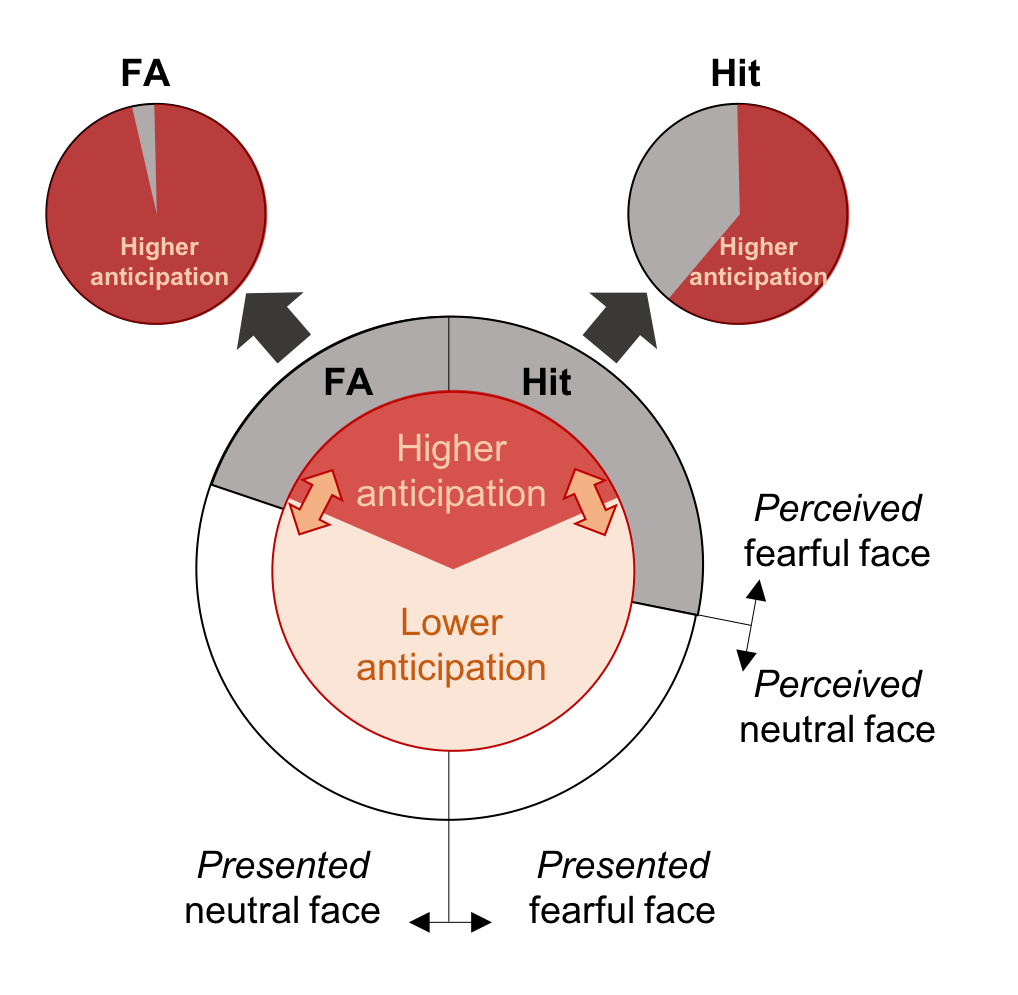

Supplement: Extended Data Figure 1-1 — Illustration of the differential contribution of anticipation for the HIT and FA trials. Given that there was no trial-wise cue to forecast the upcoming face stimulus (i.e., fearful or neutral), it is expected that anticipation for the fearful face target was similarly fluctuating across all the trials regardless of the actual type of face stimulus. That is, regardless of the presented face stimulus (fearful or neutral), there are likely to be similar proportions of trials with relatively higher level of anticipation (see inner red circle in the center). When comparing the HIT and FA trials, however, heightened anticipation is likely to contribute to a larger proportion of the FA trials relative to the HIT trials where sensory inputs of presented fearful face also contributed to the percept. This can be inferred when simply considering the proportion of HIT trials that are mainly driven by sensory inputs or alternatively by anticipation. That is, while the proportion of HIT trials (≒55%) that is the same as the total FA rate (≒25%) may be attributed to amplified anticipatory processing, the remaining HIT trials (≒30% out of 55%) is likely to reflect sensory inputs for the target faces instead. Thus, FA trials are more likely to reflect top-down-related processing than are HIT trials. Here, trials are binary divided into higher and lower anticipation trials for simplicity, and the actual proportions may vary across participants. Download Figure 1-1, PNG file. [file sup_enu-eN-NWR-0429-19-s01.png]

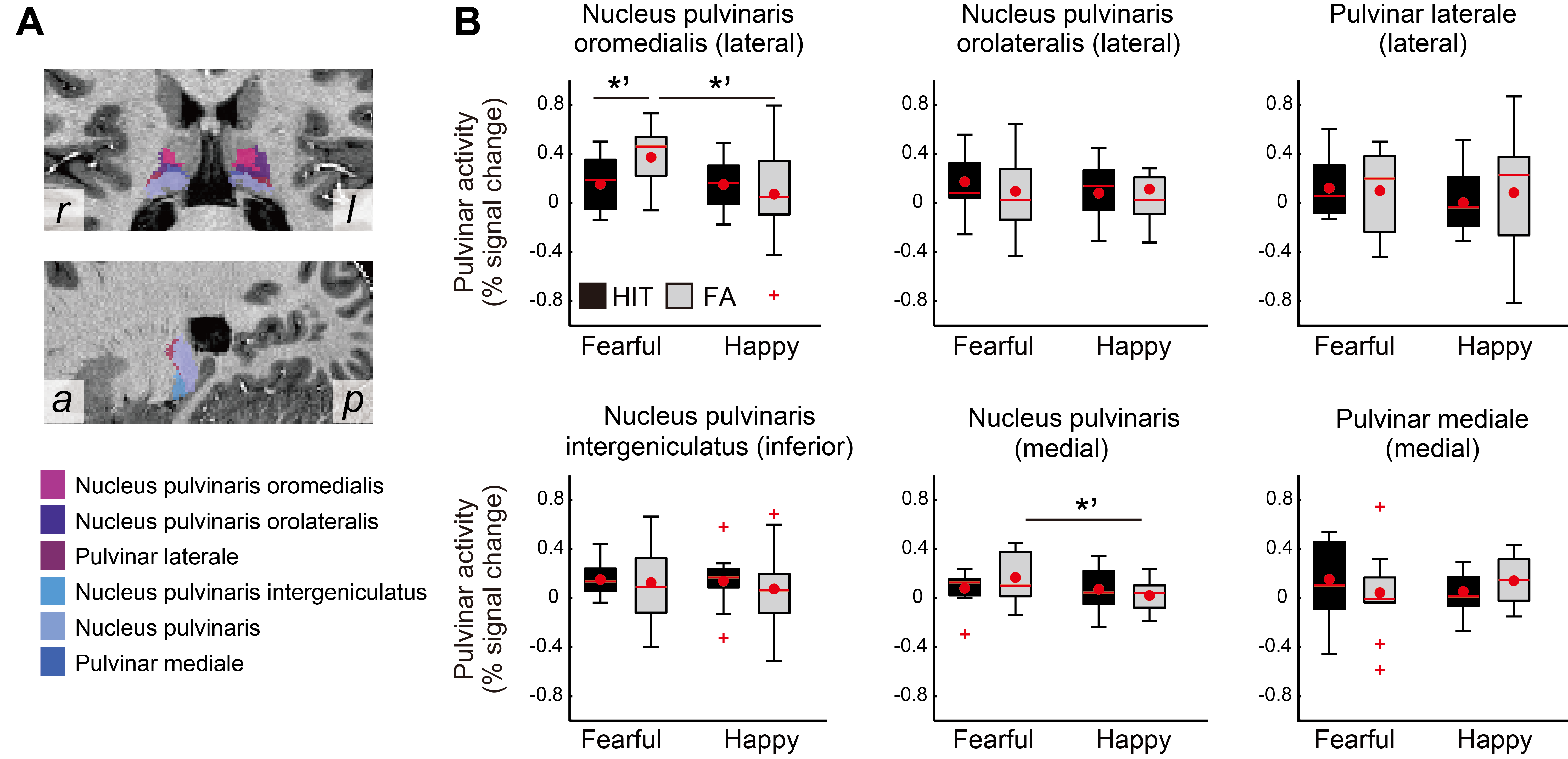

Supplement: Extended Data Figure 2-2 — Activity of the pulvinar on FA and HIT trials in each of its subregions. A, The subregions composing the lateral, inferior, and medial portions of the pulvinar were defined based on a histological atlas (Chakravarty MM et al. 2006). The atlas was imported to BrainVoyager, and the entire pulvinar including all subregions was manually aligned to the pulvinar in each participant’s Native space. We included the subregions that compose the lateral, inferior, and medial portions of pulvinar which are widely implicated in visual processing (Pessoa and Adolphs 2010; Bridge et al. 2016), namely the nucleus pulvinaris oromedialis (lateral), nucleus pulvinaris orolateralis (lateral), pulvinar laterale (lateral), nucleus pulvinaris intergeniculatus (inferior), nucleus pulvinaris (medial), and pulvinar mediale (medial). The entire voxels within each subregion were used to estimate the activity level. B, Similarly to the results in the main text (Fig. 2) with the functionally defined pulvinar ROIs, there was significantly larger activity on the FA trials of a fearful face relative to the FA trials of a happy face in two subregions, namely the nucleus pulvinaris oromedialis (lateral) and nucleus pulvinaris (medial). However, there was no significant interaction between emotion (fearful/happy) and condition (HIT/FA) in these two subregions (nucleus pulvinaris oromedialis: F(1,10) = 4.496, p = 0.060; nucleus pulvinaris: F(1,10) = 2.327, p = 0.158). The results suggested that one lateral and one medial subregion showed significantly larger activity on FA trials of a fearful face relative to FA trials of a happy face. Given that the same neutral faces were presented on these trials, the difference in activity is likely to be due to the perceived emotion driven by anticipation of a fearful versus happy face target. These results hint at the possibility that task-driven anticipation of threat signals may be coded in the medial pulvinar through its interaction with the prefrontal areas w [file sup_enu-eN-NWR-0429-19-s03.tif]

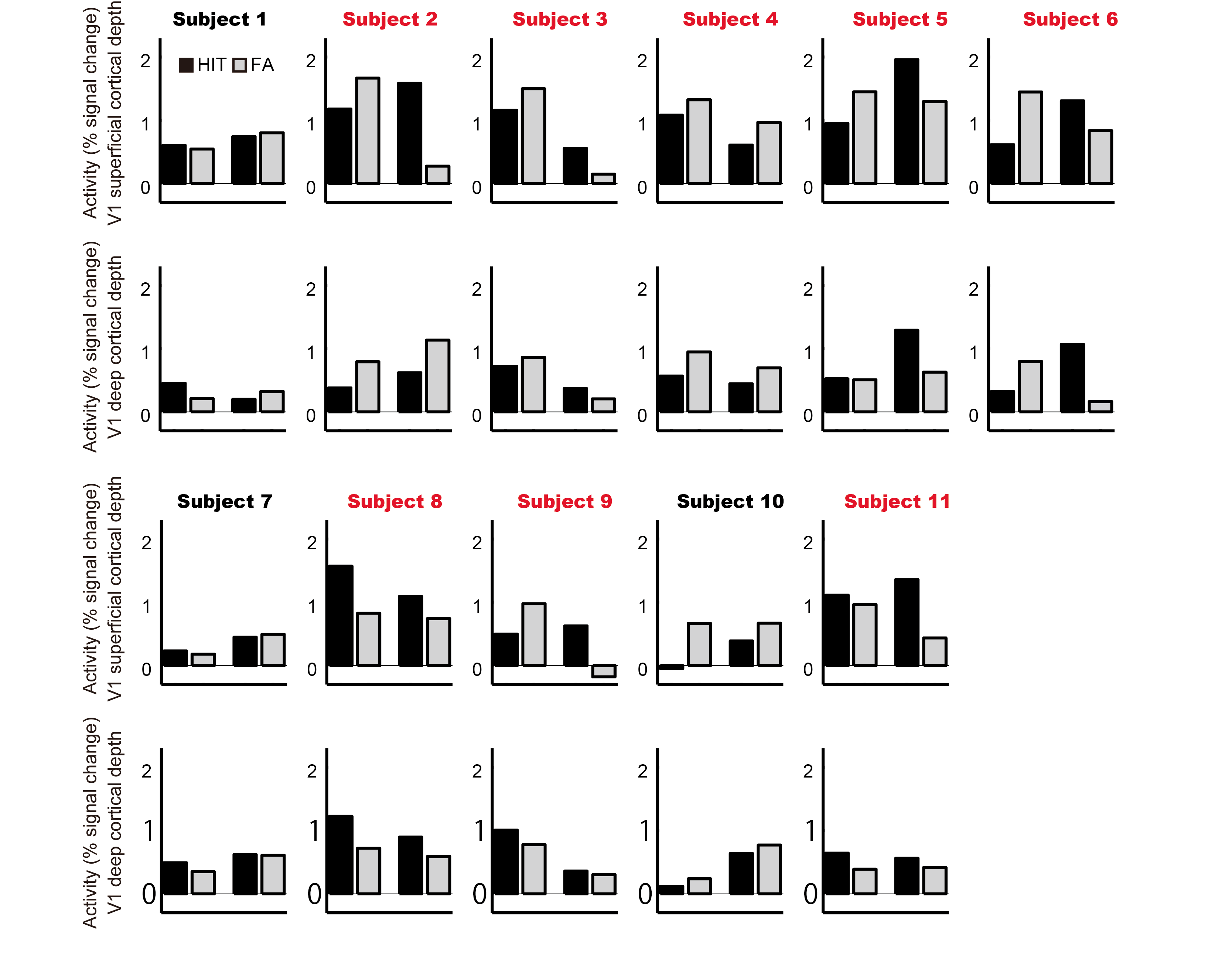

Supplement: Extended Data Figure 3-1 — V1 activity in each participant at superficial (top row) and deep cortical depths (bottom row). For each subject, deconvolved time course of V1 during the HIT and FA trials during the fearful face detection task and happy face detection task (left and right panels, respectively) are shown. Subjects who showed numerically larger activity on FA trials of a fearful face relative to FA trials of a happy face, which is in line with the pattern of group level result shown in Figure 3C, are highlighted with red fonts (eight out of 11 participants). Related to Figure 3. Download Figure 3-1, TIF file. [file sup_enu-eN-NWR-0429-19-s04.tif]

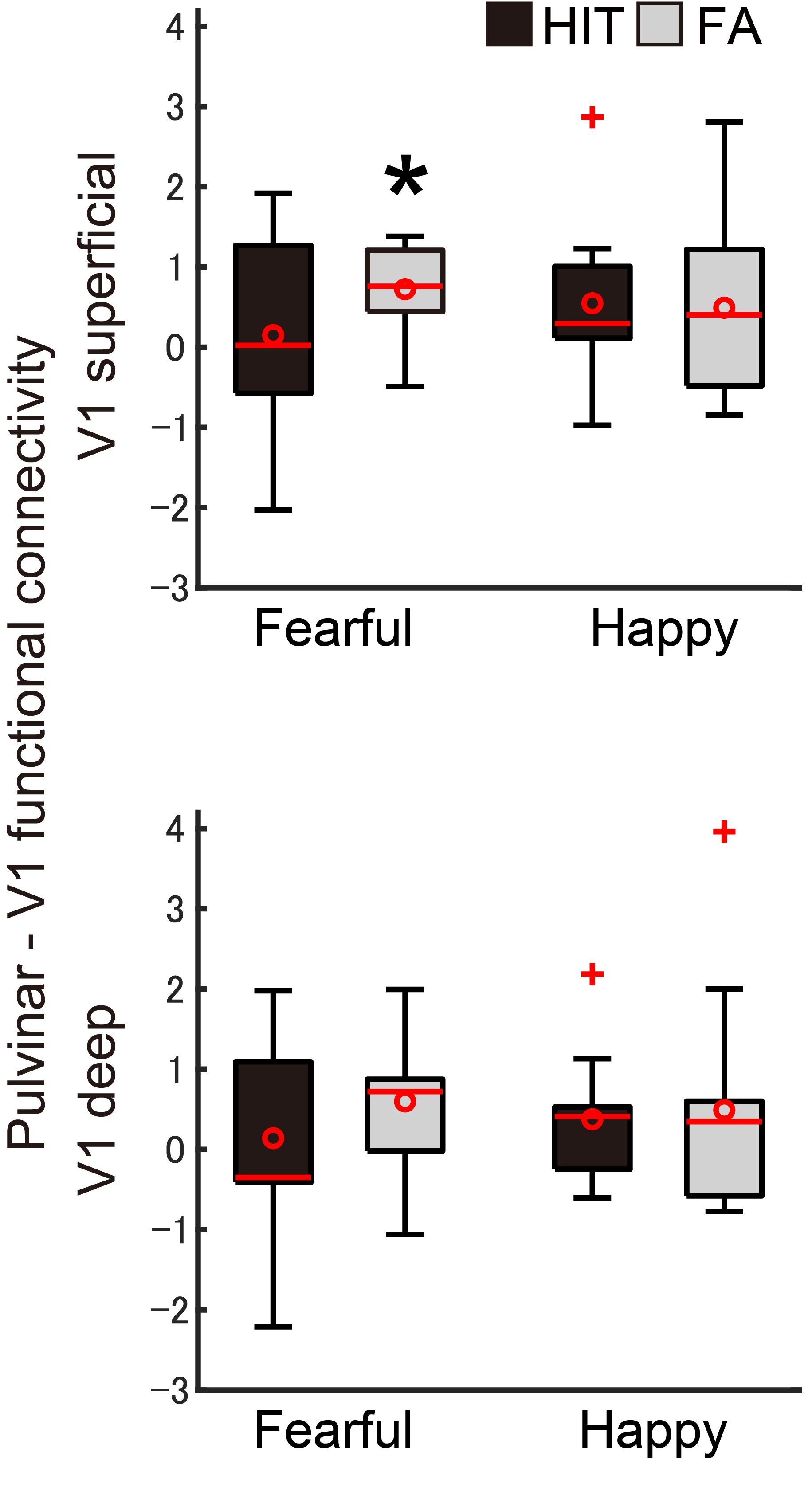

Supplement: Extended Data Figure 3-2 — The results of gPPI analyses examining the connectivity between the pulvinar and V1. gPPI analyses were separately conducted, with the V1 superficial or deep cortical depth voxels as the seed ROI [see Materials and Methods, Generalized form of context-dependent psychophysiological interaction analysis (gPPI)]. Mean t values for the parameter estimates in gPPI for each trial type and seed ROI are shown. Only during FA trials with fearful faces, a significant modulation of connectivity was present between the pulvinar and V1 superficial layers (t(10) = 3.981, p = 0.0026, one-sample t test against 0, significant after Bonferroni correction). We note that these results are only indicative, as there was no significant interaction between conditions (HIT/FA), facial emotion (fearful/happy), and V1 cortical depth (superficial/deep; F(1,10) = 0.198, p = 0.666). There was no evidence that the connectivity between the pulvinar and V1 was modulated during other conditions in either V1 cortical depths. With the V1 superficial cortical depth as a seed ROI, fearful HIT: t(10) = 0.403, p = 0.695, CI [-0.683, 0.985]; fearful FA: t(10) = 3.981, p = 0.0026, CI [0.319, 1.128]; happy HIT: t(10) = 1.869, p = 0.091, CI [–0.105, 1.120]; happy FA: t(10) = 1.4364, p = 0.181, CI [–0.271, 1.253]. With the V1 deep cortical depth as a seed ROI, fearful HIT: t(10) = 0.398, p = 0.699, CI [–0.641, 0.92]; fearful FA: t(10) = 2.223, p = 0.05, CI [–0.001, 1.199]; happy HIT: t(10) = 1.594, p = 0.142, CI [–0.15, 0.01]; happy FA: t(10) = 1.152, p = 0.276, CI [–0.459, 1.441]. We note that the null results do not necessarily indicate the absence of effects, because a PPI analysis is generally low in power (O'Reilly et al., 2012) and tSNR in the pulvinar was relatively low in the current study. Future studies may further examine the potential changes in the functional connectivity with the pulvinar across the V1 cortical depths and experimental conditions. pulv: pulvinar, a: anterior, p: posterior. Downloa [file sup_enu-eN-NWR-0429-19-s05.tif]

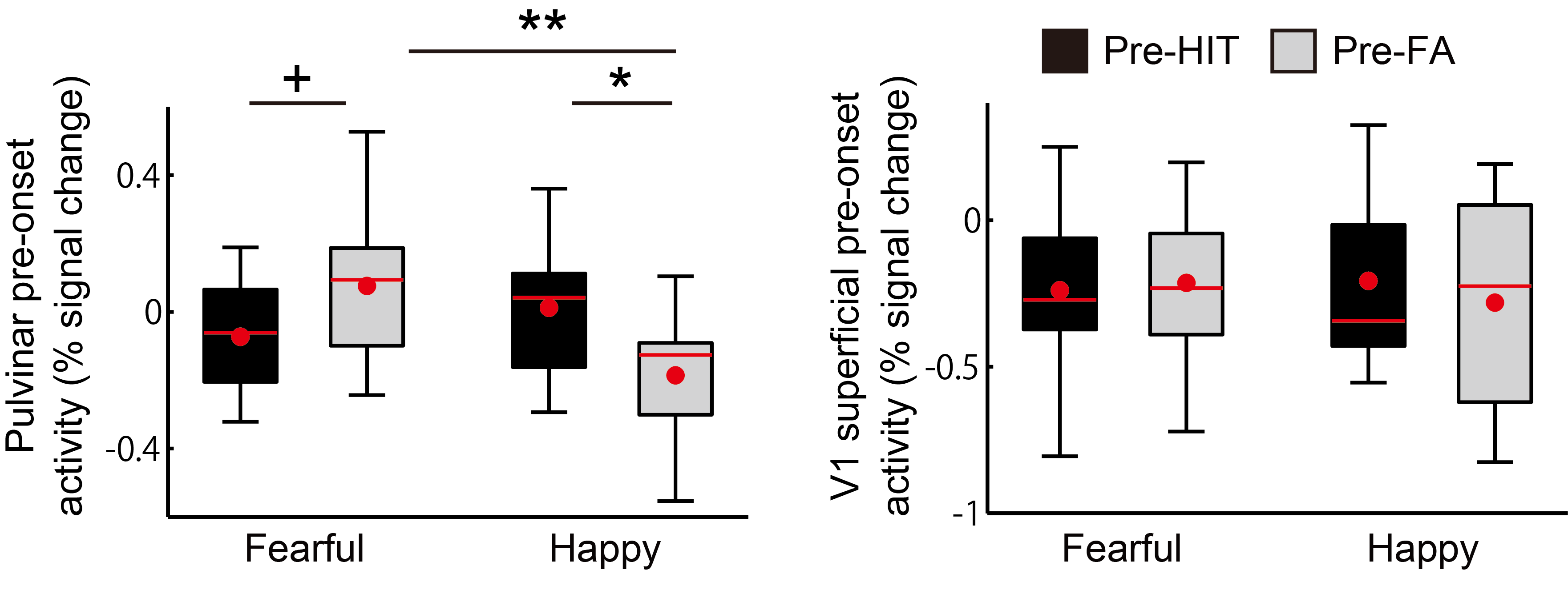

Supplement: Extended Data Figure 3-3 — Prior to the onsets of target faces, there was already enhanced activity in the pulvinar preceding the FA trial of a fearful face compared with a happy face (left panel). To estimate activity prior to the face onsets, the z-normalized signal change (%) in the preprocessed raw time course (see Materials and Methods, fMRI processing) was averaged between the two time points immediately before the face onsets (i.e., –5 and –2.5 s) relative to the preceding baseline (averaged between the two earlier time points, i.e., –10 and –7.5 s). A repeated-measures ANOVA with two factors of percept type (pre-HIT/pre-FA) and emotion (fearful/happy) revealed a significant interaction (F(1,10) = 10.094, p = 0.010). Post hoc analyses revealed that pulvinar activity was significantly greater on FA trials of fearful faces compared with happy faces in the pre-onset period (t(10) = 3.392, p = 0.007, d = 1.1). During the same pre-onset period, while pulvinar activity was significantly larger on HIT than on FA trials of happy faces (t(10) = 2.386, p = 0.038, d = 0.7), there was a non-significant opposite trend such that activity was relatively greater on FA than on HIT trials of fearful faces (t(10) = –2.147, p = 0.057, n.s.). Meanwhile, in the same pre-onset period, there was not yet any differential activity in the V1 superficial cortical depth (right panel), with no significant main effects or interaction between percept type and emotion (ps > 0.50, n.s.). These results suggest that anticipatory activity in the pulvinar modulated the response of the V1 superficial cortical depth triggered by the subsequent onset of a face target shown in Figure 3D. Box plot shows upper (75%) and lower (25%) quartiles with median (red line) and mean (red dot), with whisker showing maximum and minimum value; **p < 0.01, * p < 0.05, +p < 0.10. Related to Figures 2, 3. Download Figure 3-3, TIF file. [file sup_enu-eN-NWR-0429-19-s06.tif]

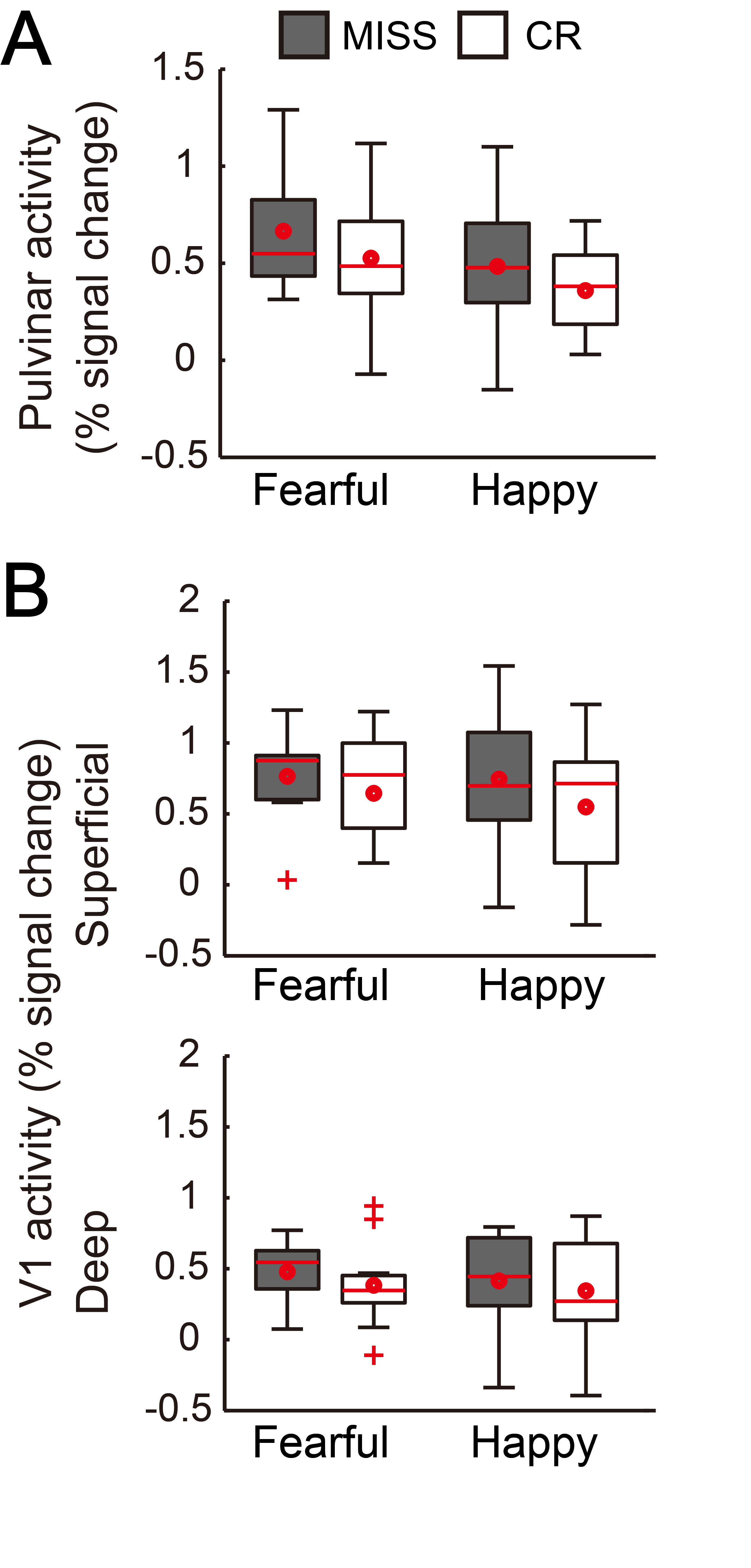

Supplement: Extended Data Figure 3-4 — The results of a control analysis showing no differential activity in the pulvinar and V1 on MISS versus CR trials. We examined whether similar results on FA trials of fearful faces (Figs. 3B, 4D) may be present on MISS trials, where a fearful face was presented but was not detected. While the results for FA trials of fearful faces may reflect enhanced top-down processing, other non-mutually exclusive possibilities are worth considering. Specifically, the results for FA trials may reflect a mere mismatch between sensory input and reported percept. Although this possibility is unlikely considering the fact that such results did not generalize to FA trials of happy faces, there remains a possibility that such input-to-percept mismatch may evoke certain neural activity only in anticipation of threat cues. We therefore examined whether the patterns of results we obtained from the contrast between FA and HIT trials may be also obtained from the contrast between MISS and CR trials, where neutral faces were perceived with or without mismatched sensory input, respectively. A, Unlike the contrast of pulvinar activity between the HIT and FA trials shown in Figure 2, there was no difference in pulvinar activity between the MISS and CR trials regardless of the facial emotion [a non-significant interaction between percept type (MISS/CR) and emotion: F(1,10) = 0.013, p = 0.912]. Although we saw a trend for pulvinar activity to be larger for the fearful face detection task than for the happy face detection task, there was no significant main effect of emotion (F(1,10) = 2.365, p = 0.155). B, Similarly, unlike the contrast of V1 activity between the HIT and FA trials shown in Figure 3, there was no differential activity in V1 between the MISS and CR trials, regardless of emotion and cortical depth (a non-significant second-order interaction: F(1,10) = 1.251, p = 0.290). While there was generally greater activity on MISS trials relative to CR trials that was not specific to any faci [file sup_enu-eN-NWR-0429-19-s07.tif]
